# CO-loaded hemoglobin/EGCG nanoparticles functional coatings for inflammation modulation of vascular implants

**DOI:** 10.1093/rb/rbae148

**Published:** 2024-12-20

**Authors:** Sui Wu, Ruichen Dong, Yinhong Xie, Wenhao Chen, Wei Liu, Yajun Weng

**Affiliations:** Key Laboratory of Advanced Technologies of Materials, Ministry of Education, Southwest Jiaotong University, Chengdu 610031, China; School of Materials Science and Engineering, Southwest Jiaotong University, Chengdu 610031, China; Institute of Biomedical Engineering, College of Medicine, Southwest Jiaotong University, Chengdu, Sichuan 610031, China; Key Laboratory of Advanced Technologies of Materials, Ministry of Education, Southwest Jiaotong University, Chengdu 610031, China; Key Laboratory of Advanced Technologies of Materials, Ministry of Education, Southwest Jiaotong University, Chengdu 610031, China; School of Materials Science and Engineering, Southwest Jiaotong University, Chengdu 610031, China; Institute of Biomedical Engineering, College of Medicine, Southwest Jiaotong University, Chengdu, Sichuan 610031, China; Key Laboratory of Advanced Technologies of Materials, Ministry of Education, Southwest Jiaotong University, Chengdu 610031, China; Institute of Biomedical Engineering, College of Medicine, Southwest Jiaotong University, Chengdu, Sichuan 610031, China; Key Laboratory of Advanced Technologies of Materials, Ministry of Education, Southwest Jiaotong University, Chengdu 610031, China; Institute of Biomedical Engineering, College of Medicine, Southwest Jiaotong University, Chengdu, Sichuan 610031, China; Key Laboratory of Advanced Technologies of Materials, Ministry of Education, Southwest Jiaotong University, Chengdu 610031, China

**Keywords:** carbon monoxide, EGCG, hemoglobin, macrophage, inflammation

## Abstract

During the implantation process of cardiovascular implants, vascular damage caused by inflammation occurs, and the inflammatory process is accompanied by oxidative stress. Currently, carbon monoxide (CO) has been demonstrated to exhibit various biological effects including vasodilatation, antithrombotic, anti-inflammatory, apoptosis-inducing and antiproliferative properties. In this study, hemoglobin/epigallocatechin-3-gallate (EGCG) core-shell nanoparticle-containing coating on stainless steel was prepared for CO loading and inflammation modulation. Inspired by strong coordination ability with CO, hemoglobin nanoparticle was first prepared and encapsulated into EGCG metal-phenolic networks. A polydopamine (PDA) linking layer was then coated on 316 stainless steel, and the hemoglobin/EGCG nanoparticles were loaded with the subsequent PDA deposition. It showed that the maximum release amount of CO by the coating was 17.0 nmol/cm^2^ in 48 h. *In vitro* evaluations conducted in a simulated inflammatory environment revealed that the coating, which released CO from hemoglobin/EGCG nanoparticles, effectively mitigated the lipopolysaccharide-induced inflammatory response in macrophages. Specifically, it decreased the expression of tumor necrosis factor-α, increased the expression of interleukin-10, suppressed the polarization of macrophages toward the M1 phenotype and reduced intracellular reactive oxygen species (ROS). Furthermore, under simulated oxidative stress conditions, the coating decreased the apoptosis of endothelial cells induced by oxidative stress and down-regulated intracellular ROS levels. *In vivo* implantation results further confirmed that the coating, with its hemoglobin/EGCG nanoparticles and CO release capabilities, reduced macrophage-mediated inflammatory responses and modulated the polarization phenotype of macrophages.

## Graphical abstract

**Figure:**
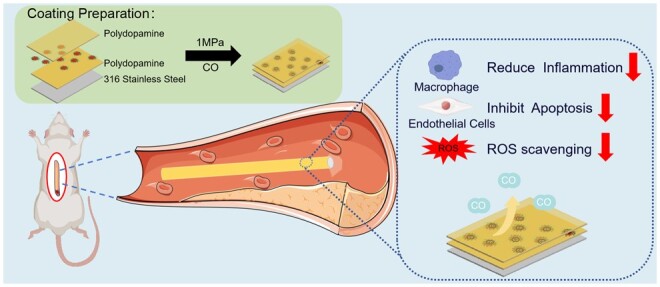

## Introduction

Carbon monoxide (CO), one of the three major gasotransmitter molecules, has been shown to possess various biological effects including vasodilation, antithrombotic, anti-inflammatory, antimicrobial, pro-apoptotic and antiproliferative actions [1–3]. Exogenous CO delivery primarily comprises inhaled CO and local delivery of carbon monoxide-releasing molecules (CORMs). However, the clinical application of inhaled CO has been constrained by challenges associated with dosage, targeting and poisoning [4]. Several CORMs have been used and studied for inflammation modulation and synergistic treatment of inflammatory diseases. So far there are still challenges such as poor stability, lack of controllability of CO release and heavy metal toxicity of CORMs for clinic applications [5].

With the rapid progress and diverse advantages of nanomedicine, a rich range of nanomaterials have been utilized for CO carrier and showed a comparably controlled CO release. The adjustable biochemical compositions, physical structure and adsorption performance have enabled the development of series of CO-releasing nanomaterials, including micelles [6–8], metal-organic frameworks (MOFs) [9–11] and proteins based nanocarriers [12]. Hemoglobin (Hb), the most abundant protein in blood, exhibits a 250 times binding capacity with CO compared with that of O_2_. Hiromi Sakai *et al*. reported a CO-loading method with hemoglobin-containing liposomes or micelles and evaluated its effects on inflammatory diseases [13–20]. It showed the benefits of CO in inflammation modulation, however, the preparation process was complex and liposomes or micelles can hardly be immobilized on coatings. Therefore, a simple and versatile CO loading method especially for coatings is needed to develop.

It is well acknowledged that chronic inflammation and oxidative stress are evolved in the major pathological mechanisms of cardiovascular diseases. Reactive oxygen species (ROS) induce oxidative stress, damage to cellular homeostasis and thus lead to cell apoptosis and tissue injure [21, 22]. Antioxidation is recognized as an effective treatment for decreasing oxidative stress and tissue injury. Metal-phenolic networks (MPNs) are supramolecular composites formed by the coordination of metal ions with phenolic ligands. It exhibits excellent antioxidant and effective ROS-scavenging properties [23]. Mei *et al*. utilized a one-pot method to synthesize Au@MPN-BMP_2_ nanoparticles for periodontitis treatment. Tannic acid (TA) and strontium (Sr^2+^) were employed to form MPN coatings on gold nanoparticles. MPN coating provides ROS scavenging capability, blocking the initiating factors of periodontitis while reducing the excessive immune response [24].

Epigallocatechin-3-gallate (EGCG) is the most abundant catechin found in green tea, showing a range of activities including antioxidant, anti-inflammatory and antibacterial properties. In the recent literature, it was utilized for antioxidation treatment including cancer, cardiovascular and neurodegenerative disease [25–27]. The pyrogallol groups in EGCG coordinates with metal ions, which form MPN coatings. MPN coatings have a strong adherence to varies substrates, showing great potential for applications in biomimetics, materials modification and biomedicine [28, 29]. In this study, hemoglobin nanoparticles (Hb-NPs) were first prepared using an anti-solvent precipitation method. Then EGCG and Fe^3+^ were used for MPN to encapsulate the hemoglobin nanoparticles (MPN@Hb-NPs). MPN@Hb-NPs was further loaded on the surface of 316 stainless steel (316LSS) by polydopamine (PDA) linker, and thus CO was loaded by pressurization. It combined the antioxidative properties of MPN and PDA and the anti-inflammatory properties of CO. The impact of the released CO on endothelial cells and macrophage behavior in a simulated inflammatory environment was thoroughly assessed *in vitro*. Furthermore, *in vivo* efficacy of the hemoglobin/EGCG nanoparticle-containing coating was further evaluated in rats.

## Materials and methods

### Materials

Rabbit Hb was acquired from Beijing Huamaike. EGCG, 2,2-diphenyl-1-picrylhydrazyl (DPPH•), and dimethyl sulfoxide (DMSO, Analytical Reagent [AR]) were from Macklin. Dopamine hydrochloride was from Aladdin. Lipopolysaccharide (LPS) was purchased from Sigma-Aldrich. Ethanol (EtOH, AR) was from Chengdu Kolon Chemical. High-purity CO gas (99.9%) was from Chengdu Shimao Gas. For ELISA analysis, tumor necrosis factor-α (TNF-α) and interleukin-10 (IL-10) kits were from Biolegend. Cell proliferation was assessed using Cell Counting Kit-8 (CCK-8) from DOJINDO. Dihydroethidium (DHE) was purchased from Jiangsu Kaiji Biology. Additionally, the Total SOD activity test kit (employing the WST-8 method) and the Mitochondrial membrane potential assay kit with JC-1 were from Beyotime. All chemicals and reagents were obtained from commercial suppliers and used without any further purification.

Eight-week-old male Sprague-Dawley rats and New Zealand White rabbits (weighing 2.5 kg) were bought from Chengdu Dashuo Laboratory Animal Co. All animals were housed in a dedicated animal facility. The experimental procedures were carried out in strict compliance with the protocols approved by the Animal Ethics Committee of Southwest Jiaotong University (SWJTU-2103-007, NSFC), ensuring adherence to the institutional ethical standards and guidelines related to animal welfare.

### Characterization

The microstructural analysis of the nanoparticles was examined using a field emission scanning electron microscope (SEM, JSM 7800F Prime) and a transmission electron microscope (TEM, JEM-2100F). Fourier transform infrared spectroscopy (FTIR, NICOLET5700 FT-IR) was employed to identify the functional groups within the samples. The UV-visible spectrophotometer (UV2600) was utilized to detect the UV-visible absorption properties. Additionally, the Malvern nanoparticle size analyzer (ZEN3600) was used to evaluate the zeta potential and particle size of the nanoparticles. The iron content was quantified via Atomic Absorption Spectrophotometry (TAS-990F). X-ray photoelectron spectrometer (Thermo Fisher Scientific ESCALAB Xi+) was utilized for Elemental composition analysis. Water contact angles were assessed using a contact angle measurement instrument (DSA100).

### Preparation of hemoglobin nanoparticles encapsulated in metal-phenolic networks (MPN@Hb-NPs) and CO loading

In brief, EtOH (3 ml, 4°C) was added dropwise to a Hb solution (2 ml, 25.0 mg/ml, 4°C), and then placed in an ice bath with continuous stirring. The pH was adjusted to around 8 using Tris buffer solution. Then 40 μl of EGCG solution (10 mg/ml) and 10 μl of FeCl_3_ solution (10 mg/ml) were added to 950 μl of the Hb suspension. Thus the suspension changed from red to reddish-brown. It was centrifuged at 8000 rpm for 5 min, and washed by three times with deionized water, and then lyophilized. The obtained nanoparticles were referred to as MPN@Hb-NPs.

**Figure 1.**
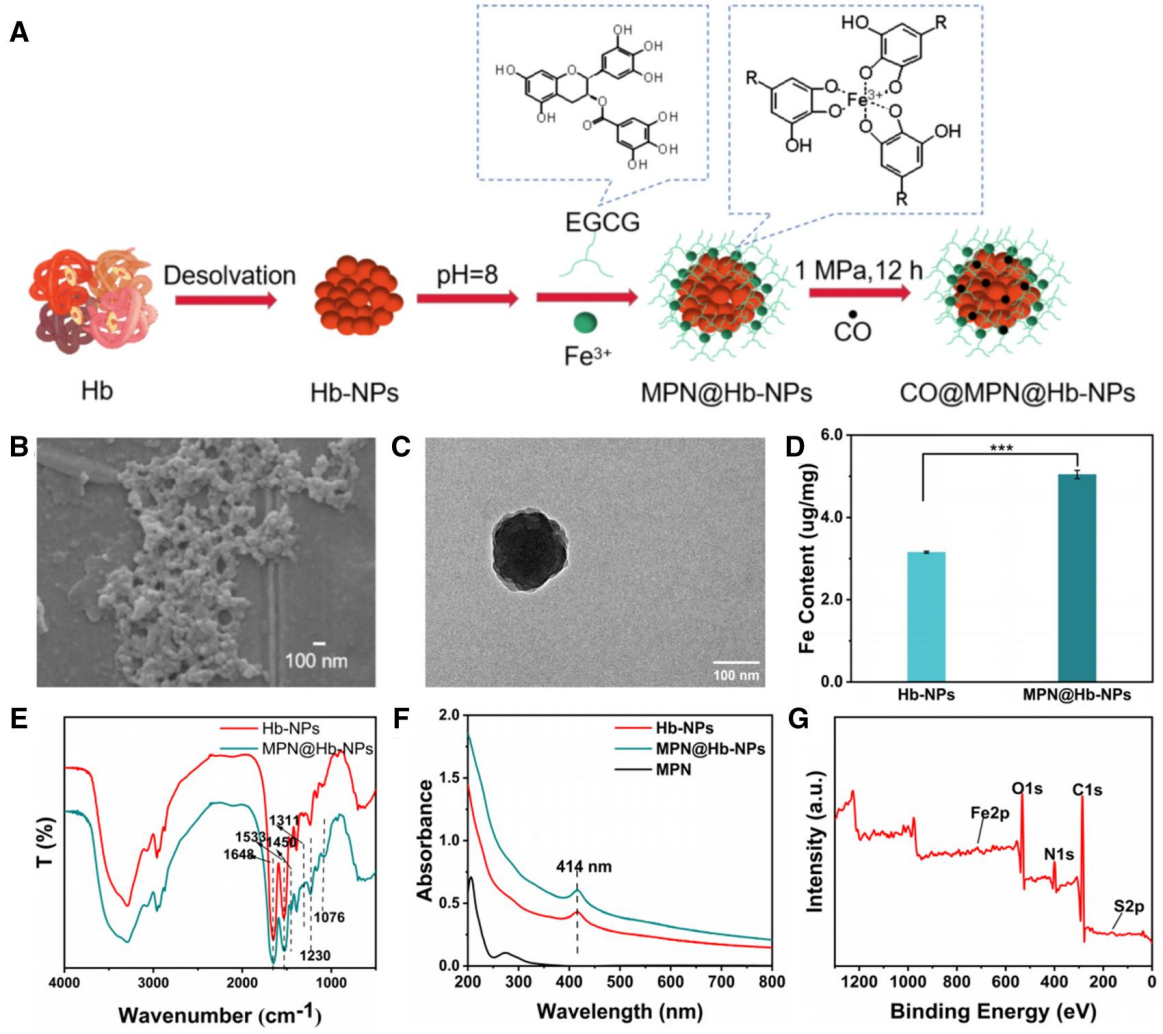
Characterization of MPN@Hb-NPs. (**A**) Schematic illustration of preparation of MPN@Hb-NPs and CO@MPN@Hb-NPs. (**B**) SEM and (**C**) TEM images of MPN@Hb-NPs. (**D**) Fe content of Hb-NPs and MPN@Hb-NPs. (**E**) FT-IR spectra of Hb-NPs and MPN@Hb-NPs. (**F**) UV-vis spectra of Hb-NPs, MPN@Hb-NPs and MPN. (**G**) XPS survey spectra of MPN@Hb-NPs. Means ± standard deviation (n = 3), P < 0.001 (***).

A specially designed CO high-pressure reaction equipment was used to load CO. The MPN@Hb-NPs samples were placed into the CO loading device, and the system was first kept in a vacuum state and then pressurized with 1 MPa of CO for 12 h. The reactor was then flushed with nitrogen, and the obtained samples were identified as CO@MPN@Hb-NPs (Figure 1A).

### Preparation of MPN@Hb-NPs coating and CO loading

316 LSS foil was cut into squares (1 × 1 cm) and was ultrasonically cleaned sequentially in ethanol and ultrapure water. After that, they were incubated in dopamine solution (2 mg/ml) in Tris buffer solution (pH 8.5) for 12 h at 37°C for PDA deposition. Then they were ultrasonically washed in water for three times. The above PDA deposition and ultrasonic washing process was repeated twice, and thus the obtained samples were labeled as PDA.

PDA samples were incubated in a solution of MPN@Hb-NPs (1 mg/ml, pH 8.5) for 24 h at 37°C. Then they were ultrasonically washed in ultrapure water for three times. The obtained samples with MPN@Hb-NPs loaded coating were labeled as HC.

To prepare a sandwich coating with PDA at the outermost layer. HC samples were incubated in dopamine solution (2 mg/ml, pH 8.5) for 12 h at 37°C. Then they were ultrasonically washed in ultrapure water for three times. The obtained samples were labeled as HC@PDA.

**Figure 2.**
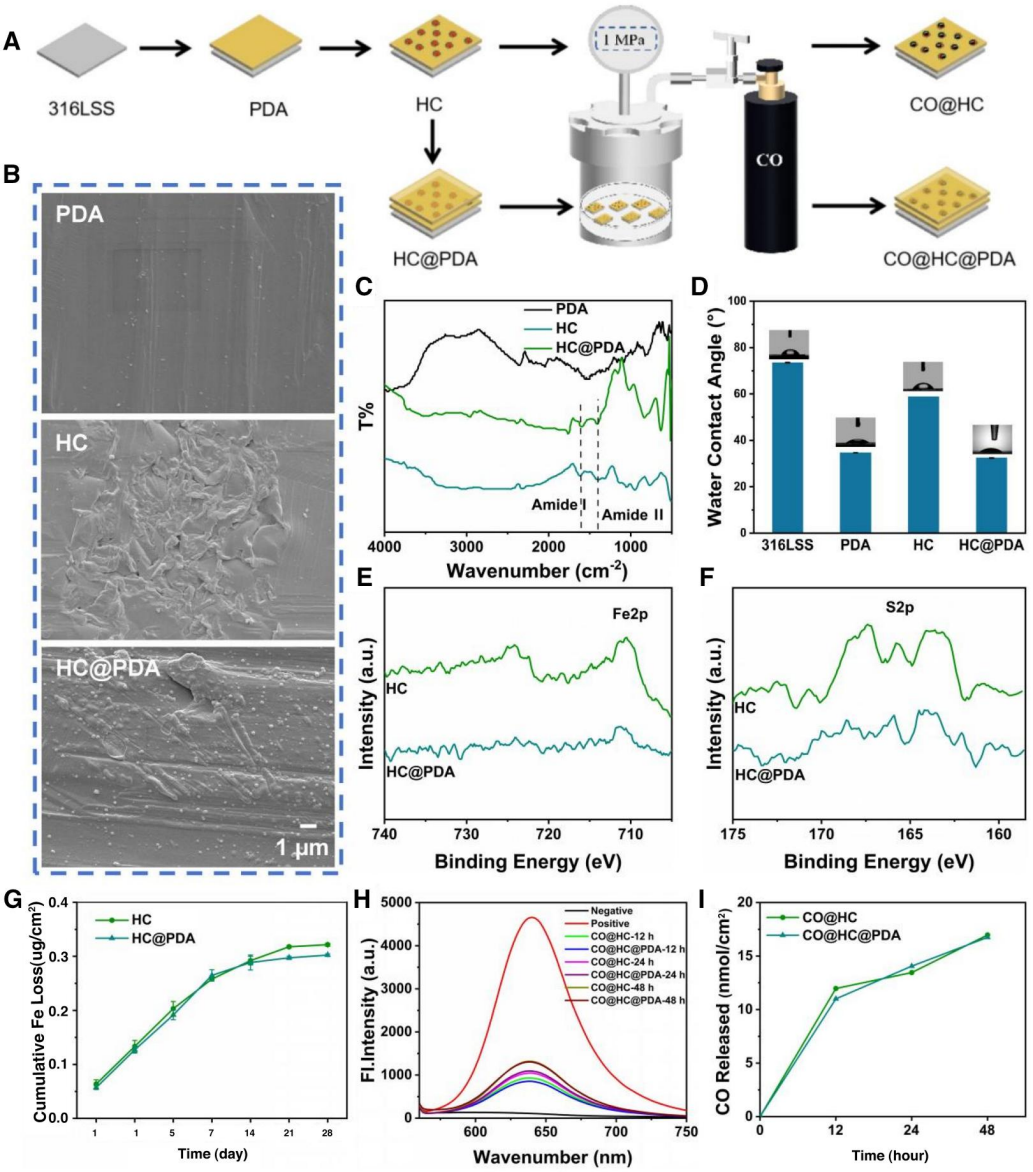
Characterization of the prepared coatings. (**A**) Schematic illustration of preparation of the prepared coatings. (**B**) SEM images of PDA, HC and HC@PDA. (**C**) FT-IR spectra of PDA, HC and HC@PDA. (**D**) Water contact angles of 316LSS, PDA, HC and HC@PDA. XPS high-resolution spectrum of elemental Fe (**E**) and S (**F**) on HC and HC@PDA. (**G**) Cumulative Fe loss of HC and HC@PDA. (**H**) fluorescence plots of 1-Ac detection of 12, 24 and 48 h CO release from CO@HC and CO@HC@PDA. (**I**) CO release from CO@HC and CO@HC@PDA at different times.

CO was loaded in the coating by pressurization with the above methods of section Preparation of hemoglobin nanoparticles encapsulated in metal-phenolic networks (MPN@Hb-NPs) and CO loading. HC-loaded CO was labeled as CO@HC, HC@PDA-loaded CO was labeled as CO@HC@PDA (Figure 2A).

### Analysis of CO release and free-radical scavenging

Based on our preliminary research, 1-Ac was used for detecting CO release of CO@MPN@Hb-NPs, CO@HC and CO@HC@PDA [10]. Following the subsequent addition of 400 μl of DMSO, 20 μl of 1-Ac (0.15 mg/ml) and 600 μl of phosphate buffer solution (PBS), the samples were incubated in an oscillator at 37°C for predetermined periods of time in dark. Ultimately, a fluorescence spectrophotometer was used to detect the solution.

DPPH• (200 μl, 0.05 mg/ml in EtOH) was mixed with samples in a 24-well or 96-well plate to assess scavenging activity. The combination was left in the dark at room temperature for half an hour. After the incubation, a plate reader was used to measure the absorbance (Abs) at 517 nm. The negative control was EtOH only, whereas the positive control was a solution of EtOH and DPPH. DPPH• Scavenging activity = ($\frac{A_{0}-A_{S}}{A_{0}}$) × 100% (*A*_0_: the Abs of DPPH•, *A_S_*: the Abs of samples).

•OH scavenging was further evaluated. The Rhodamine B (RhB) degrades with •OH increase and it will be bleached with degradation. To evaluate •OH scavenging, the change of Abs at 553 nm was observed after a half-hour reaction. The change in absorbance of the system after addition of PDA, HC and HC@PDA was determined and defined as the expression • OH scavenging rate:

$$S=(A_{s}-A_{0})/(A-A_{0})\times 100\%.$$

where *A_S_*: the absorbance of RhB with H_2_O_2_ and Fe^2+^ and samples, *A*_0_: the absorbance of RhB with H_2_O_2_ and Fe^2+^, and *A*: the absorbance only with RhB.

### *In vitro* safety and blood compatibility evaluation

*In vitro* cytotoxicity of 316LSS, PDA, HC and HC@PDA was evaluated with endothelial cells by CCK-8 assays. Briefly, the UV-sterilized samples were placed in 24-well plates, followed by seeding endothelial cells onto the samples at a density of 1 × 10^4^ cells/ml. After being incubated at 37°C with 5% CO_2_ for 24 and 72 h, the media were substituted with CCK-8-containing culture medium, and a microplate reader was used to measure cell activity.

The hemolysis rate of the samples was evaluated. Fresh anticoagulated blood was obtained from New Zealand White rabbits. Fresh anticoagulated rabbit blood was centrifuged at 3000 rpm for 15 min, and then red blood cells were collected for subsequent hemolysis experiments. Some red blood cells were diluted to 2% with ultrapure water as the positive control and some were diluted with saline as the negative control. The samples were placed in 24-well plates and the saline diluted red blood cells (2%) were added. All samples were incubated in a shaker at 37°C for 1 h. The medium was collected and centrifuged at 3000 rpm for 5 min. The supernatant was collected and Abs of 545 nm was detected by an microplate reader.

### *In vitro* evaluation of modulation of macrophage inflammation with LPS

The UV-sterilized samples were placed in 24-well plates, then macrophages were seeded on the samples with a density of 5 × 10^4^ cells/ml and cultured with 500 ng/ml LPS. After 24 h of culture, the media were substituted with CCK-8-containing culture medium, and a microplate reader was used to measure cell activity. Additionally, to quantify the levels of TNF-α and IL-10 cytokines, cell supernatants were collected and analyzed using Mouse IL-10 ELISA and Mouse TNF-α ELISA kits, respectively.

The immunofluorescence staining protocol for phenotypic assessment was executed as detailed below. After a 24-h incubation period, the supernatant was removed. The treated macrophages were subjected to washing with PBS thrice, fixed with glutaraldehyde and subsequently blocked with 10% bovine serum albumin (BSA). Following this, immunofluorescence staining was performed utilizing anti-CD197 antibodies. After an additional washing with PBS thrice, the appropriate secondary antibodies were introduced, and the nuclei were stained with 4',6-diamidino-2-phenylindole (DAPI) for visualization.

### Evaluation of injured endothelial cells in a simulated ROS-containing microenvironment

A similar *in vitro* endothelial cell injury evaluation method was used as reported in our previous study [30]. Details were as follows: endothelial cells were seeded onto the UV-sterilized samples at a density of 2 × 10^4^ cells/ml and cultivated for 24 h in 24-well plates. Subsequently, a common ROS inducer H_2_O_2_ was added to the cultures with a final concentration of 200 μM and then cultured for another 4 h. During this period, cell apoptosis coincided with alterations in mitochondrial membrane potential was assessed. In brief, the cells co-treated with the samples and H_2_O_2_ were washed with PBS and stained with JC-1 for 20 min. After that, they were rinsed with JC-1 buffer to prepare for examination. Additionally, the ROS level was analyzed through DHE staining. Specifically, the cells co-treated with the samples and H_2_O_2_ were washed with PBS and stained with DHE for 30 min. After being washed, the samples were visualized under a fluorescence microscope. Subsequently, the average fluorescence intensity of each sample was quantified utilizing Image J software.

### *In vivo* implantation

Eight-week-old male Sprague-Dawley rats were divided into three groups, namely, 316LSS, PDA and CO@HC@PDA (*n* = 3 per group). Rats were anesthetized and the prepared samples were implanted into the abdominal aorta. After 30 days of implantation, the vessels were removed at the sample location, rinsed with saline, fixed with 4% paraformaldehyde overnight and sectioned by paraffin embedding. Soft tissue sections were stained with HE immunofluorescence staining, CD68 immunofluorescence staining.

### Statistical analysis

The data are presented as means ± standard deviation (*n* = 3) and were analyzed using one-way ANOVA. Significance levels are indicated as follows: *P* < 0.05 (*), *P* < 0.01 (**), and *P* < 0.001 (***).

## Results

### Characterization of MPN@Hb-NPs

Hb-NPs were prepared via desolvation precipitation in EtOH under ice bath conditions. MPN shell was obtained via the crosslinking of EGCG and Fe^3+^ (Figure 1A). Based on the SEM results, the as-prepared MPN@Hb-NPs exhibited a uniform nanospherical morphology (Figure 1B). The morphologies of MPN@Hb-NPs were examined using TEM, presenting a clear spherical core-shell nanostructure (Figure 1C). The hydrodynamic diameter of MPN@Hb-NPs was 422 nm with a low polydisperse index of 0.19 and high ζ-potential of 19.6 (Supplementary Figure S1 and Table S1), compared with that of Hb-NPs of 0.20 and 4.89, respectively.

Figure 1E depicted the FTIR spectra of Hb-NPs and MPN@Hb-NPs, clearly exhibiting the distinctive bands of Amide I at 1648 cm^−1^ and Amide II at 1533 cm^−1^. Additionally, it revealed the corresponding bands for the phenolate groups of the aromatic rings of EGCG at 1311 and 1076 cm^−1^ specifically on MPN@Hb-NPs. The absorption peak at 1230 cm^−1^ indicates the interaction of phenolate groups with the Fe^3+^ center. Also, the absorption peak at 1450 cm^−1^ could further be attributed to the stretching vibration of the benzene ring skeleton. Thus, the FTIR spectra of MPN@Hb-NPs were in accord with the MPN and Hb chemical composition. In addition, Hb also has a characteristic UV-vis Abs spectrum of the heme group [31]. Figure 1F shows the UV-vis spectra of MPN, Hb-NPs and MPN@Hb-NPs. The Hb-NPs and MPN@Hb-NPs displayed the characteristic peaks of the heme porphyrin ring structure at 414 nm, while the MPN did not show the characteristic peak at 414 nm. It indicated that the heme structure of Hb did not change in the preparation process of desolvation and MPN encapsulating. Atomic absorption spectrophotometer detection results showed a significant difference in iron content between MPN@Hb-NPs and Hb-NPs (Figure 1D). The chemical composition of MPN@Hb-NPs was also analyzed by XPS. As shown in the survey spectra of** **Figure 1G, C, N, O, Fe and S were detected. The high-resolution spectrum of elemental Fe and elemental S were shown in Supplementary Figure S2. Supplementary Table S2 shows the elemental percentage of MPN@Hb-NPs, and the content of Fe is 0.5%. This result is consistent with the result of 5 μg of Fe per milligram of particles measured by atomic absorption.

### CO release and DPPH• scavenging of MPN@Hb-NPs

CO release of CO@MPN@Hb-NPs was detected using a CO fluorescent probe 1-Ac. Palladium (II) bind to CO once it is released from the samples, causing 1-Ac to dissociate and fluorescent Nile Red to be released [10]. CO gas was used as the positive control, and it showed that the fluorescence intensity was significantly enhanced when CO was added in 1-Ac. To investigate the duration of CO release, CO@MPN@Hb-NPs were incubated in 1-Ac for 12, 24, 36 and 48 h, respectively and then the remained CO release was detected. The results showed that the remained CO release increased with the incubation time, and the fluorescence intensity was much greater than that of the negative control (Supplementary Figure S3A).

To investigate the mechanism of CO loading in MPN@Hb-NPs, Hb-NPs were replaced with BSA-NPs to prepare MPN@BSA-NPs. Additionally, Hb-NPs and MPN were also prepared. From Supplementary Figure S3B, it can be observed that the MPN can load CO, but it released rapidly with nearly complete release within 12 h. The higher fluorescence intensity observed in MPN@BSA-NPs compared to MPN may be attributed to the large surface area and physical adsorption of CO, and thus it extended the release duration to 24 h compared to that of MPN. Hb-NPs loaded CO through chemical binding with the porphyrin structure in Hb. So it showed the release was continued beyond 36 h. There was more CO released from MPN@Hb-NPs than that of Hb-NPs in 36 h. Based on the fluorescence intensity of positive controls, the CO release from MPN@Hb-NPs is about 22.8 nmol/mg (Supplementary Figure S3C) with a release duration of 36 h.

Fe-based MOFs were used for CO loading due to their highly ordered porous structure and Fe center. MOFs are a type of MPN, sharing a similar composition where metals coordinate with organic molecules, which suggests a possibly similar CO loading mechanism of coordination binding and physical loading. For the MPN preparation, in this study, Fe^3+^ was used and it had weak coordination ability with CO. So it showed a rapid and low CO release of MPN from fluorescence intensity detection.

For MPN@Hb-NPs, the binding of CO to Hb-NPs is mainly through high coordination affinity, and the binding of CO to MPN is through low coordination affinity and physical loading. In conclusion, the synthesized MPN@Hb-NPs particles in this study load CO through both physical and chemical binding mechanisms.

DPPH• is a comparable stable radical with purple color. When it reacts with antioxidant, DPPH• accepts an electron, turning colorless as DPPH-H. It was used to evaluate the ability of samples to scavenge free radicals. Supplementary Figure S3D illustrated the DPPH• scavenging rates of MPN@Hb-NPs and CO@MPN@Hb-NPs with different concentrations (1, 5 and 10 mg/ml). The scavenging rate of DPPH• increased with the concentration, which mainly attributed to MPN’s antioxidative properties. It showed CO loading has little impact on DPPH• scavenging rates.

### Characterization of MPN@Hb-NPs coating

The SEM results showed the irregular morphology on the surface of HC and HC@PDA compared to PDA (Figure 2B). The surface chemical composition of HC and HC@PDA samples was characterized by Fourier infrared spectra (Figure 2C). Compared to PDA, both HC and HC@PDA showed amide-amide I bands (1610 cm^−1^) and amide II bands (1410 cm^−1^), providing preliminary evidence that the particles were successfully deposited on the PDA coating. The water contact angle was 74° for sample 316LSS, 35° for sample PDA, 60° for sample HC and 35° for sample HC@PDA (Figure 2D). The hydrophilic groups amino and phenolic hydroxyl groups in PDA as well as MPN@Hb-NPs lead to a lower water contact angle.

The atomic spectrophotometer results showed that the Fe content on the surface of the HC and HC@PDA coatings was about 2 μg/cm^2^ (Supplementary Figure S4). HC and HC@PDA were incubated in PBS for 1, 3, 5, 7, 14, 21 and 28 days to measure the release of elemental Fe (Figure 2G). It showed the release of Fe was nearly the same during 1–7 days for both samples, and after 7 days the release of HC@PDA was slightly lower than that of HC, which may be due to the extra layer of PDA on the surface. The release of Fe from the HC@PDA in 28 days was only about 15% of the total amount of Fe, indicating that the coatings have good water stability.

The surface chemical composition of HC and HC@PDA was analyzed by XPS to evaluate their surface chemistry. Results showed that C, N, O, Fe and S were detected on the surface (Supplementary Figure S5 and Table S3). As with the MPN@Hb-NPs, the high-resolution spectrum of elemental Fe showed an absorption peak of elemental Fe 2p at 712 eV (Figure 2E), and the high-resolution spectrum of elemental S showed an absorption peak of elemental S 2p at 164 eV (Figure 2F).

Similarly, CO release of CO@HC and CO@HC@PDA was detected using a CO fluorescent probe 1-Ac. CO release of HC and HC@PDA was both detected and it showed there was little difference in both samples (Figure 2H). And the maximum amount of CO released from CO@HC and CO@HC@PDA within 48 h is 17.0 nmol/cm^2^ (Figure 2I).

### Antioxidant properties evaluation of the prepared coatings

**Figure 3.**
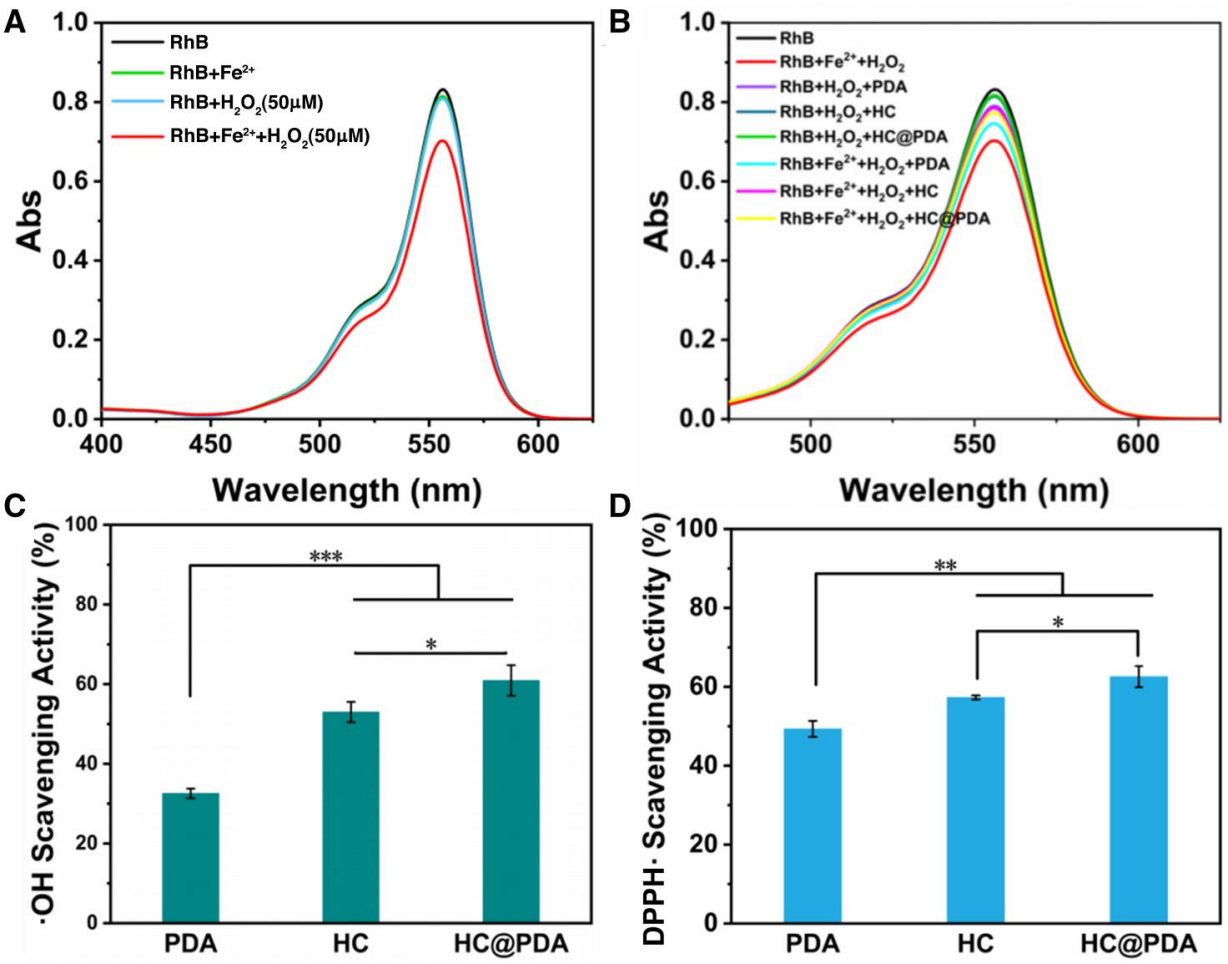
Antioxidant properties evaluation of HC@PDA. (**A**) UV−vis analysis of RhB with different Fenton reagents. (**B**) UV−vis analysis of RhB with Fenton reagents and samples. (**C**) •OH scavenging rate of samples. (**D**) DPPH• scavenging rate of samples. Means ± standard deviation (n = 3), P < 0.05 (*), P < 0.01 (**), and P < 0.001 (***).

Typically, H_2_O_2_ is found in pathological microenvironments such as atherosclerosis, with concentrations typically around several μM. Certain transition metals, including Fe, have been reported to catalyze the degradation of H_2_O_2_, leading to the simultaneous production of •OH through Fenton or Fenton-like reactions [32]. To investigate the ability of HC and HC@PDA to scavenge •OH, a RhB decolorization experiment was conducted. After half an hour of reaction, the absorption spectra of RhB solution and RhB-Fe^2+^-H_2_O_2_ solution were determined, respectively. The absorbance of RhB-Fe^2+^-H_2_O_2_ solution decreased significantly, which was a result of •OH generated by Fenton’s reagent and bleaching of RhB (Figure 3A).

When H_2_O_2_ was only introduced, RhB was not bleached by the HC and HC@PDA because no •OH generated without free Fe^2+^ (Figure 3B). When H_2_O_2_ and Fe^2+^ were introduced, RhB was slightly degraded with HC and HC@PDA in the solution (Figure 3B). These results indicated that HC and HC@PDA can scavenge •OH.

The results of the •OH scavenging rate indicated that there was a notable difference in the •OH scavenging ability of HC and HC@PDA relative to PDA (Figure 3C), indicating that MPN@Hb-NP encapsulation enhanced the ability of scavenge •OH.

DPPH• are involved in oxidative stress and may cause damage to cells and tissues. In section CO release and DPPH• scavenging of MPN@Hb-NPs, it showed that MPN@Hb-NPs had the ability to scavenge DPPH•. The same method was used to test whether HC and HC@PDA had the ability, and the results were shown in Figure 3D. It showed that PDA also had the ability to scavenge DPPH•. While the ability of HC and HC@PDA to scavenge DPPH• was significantly enhanced compared to that of PDA. There was also a significant difference between the HC and HC@PDA, which may attribute to the added PDA layer in HC@PDA.

**Figure 4.**
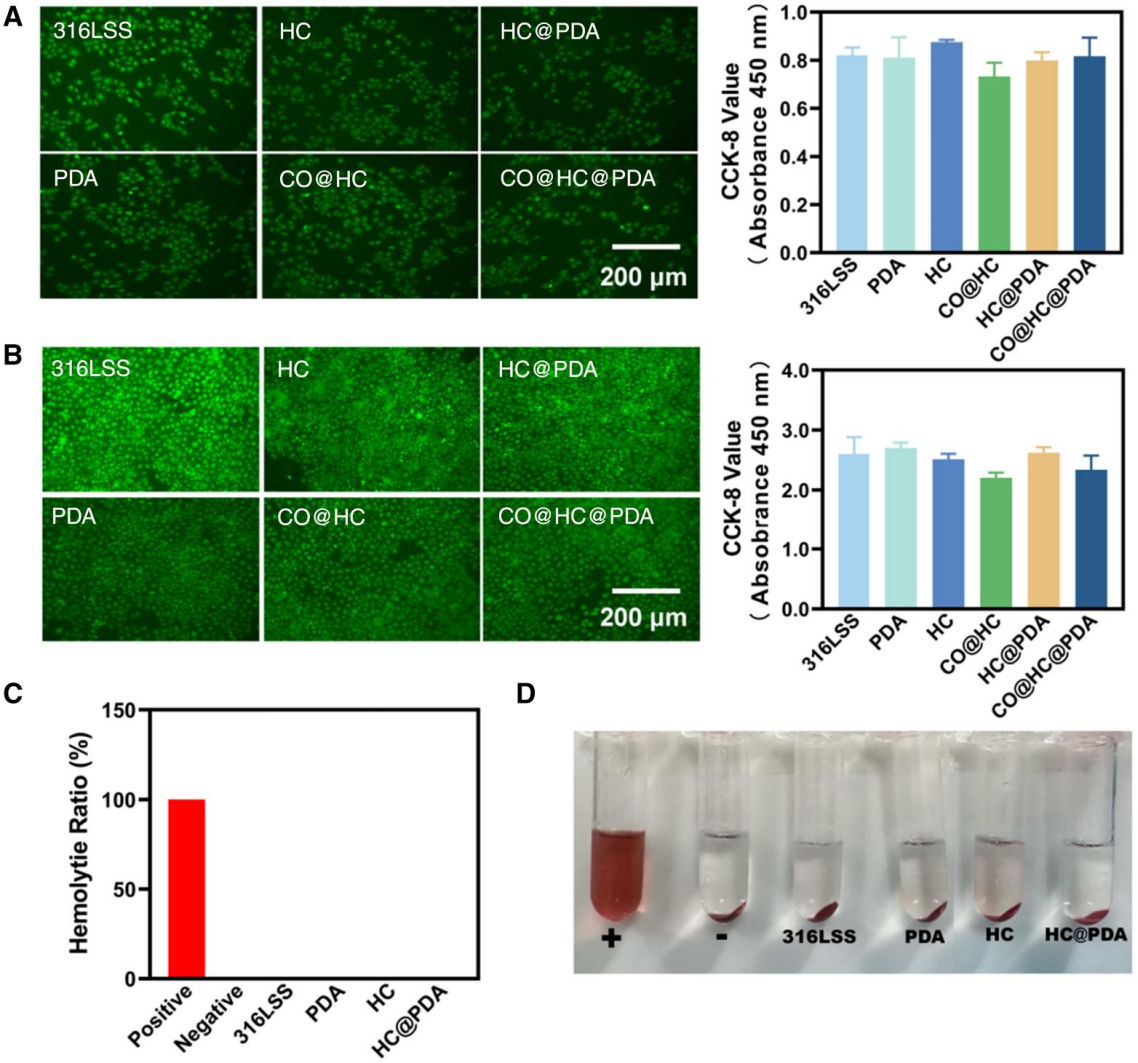
*In vitro* safety evaluation and blood compatibility evaluation. Fluorescence images and CCK-8 assay of endothelial cells after (**A**) 1 day and (**B**) 3 days of culture. (**C**) Hemolysis rate of HC and HC@PDA. (**D**) Digital photographs of the supernatant of HC and HC@PDA in contact with blood.

### *In vitro* safety evaluation and blood compatibility evaluation

Cellular safety and hemocompatibility are essential prerequisites for further application [30]. The cytotoxicity of 316LSS, PDA, HC, CO@HC, HC@PDA and CO@HC@PDA on endothelial cells (ECs) was evaluated. Samples were cultured with endothelial cells *in vitro* for 24 and 72 h, and the CCK-8 value reflect cell activity. The results demonstrated that HC, CO@HC, HC@PDA and CO@HC@PDA had no obvious cytotoxicity compared to the control after 24 and 72 h of culturing (Figure 4A and B).

The assessment of hemolysis rate is a crucial parameter to evaluate the compatibility of blood-contacting biomaterials. Therefore, the hemolysis of 316LSS, PDA, HC and HC@PDA was examined *in vitro*. The results revealed that the hemolysis rates for all materials were below 5% (Figure 4C and D), meeting the established standards for blood-contacting biomaterials.

### *In vitro* evaluation of modulation of LPS-stimulated macrophage inflammation

**Figure 5.**
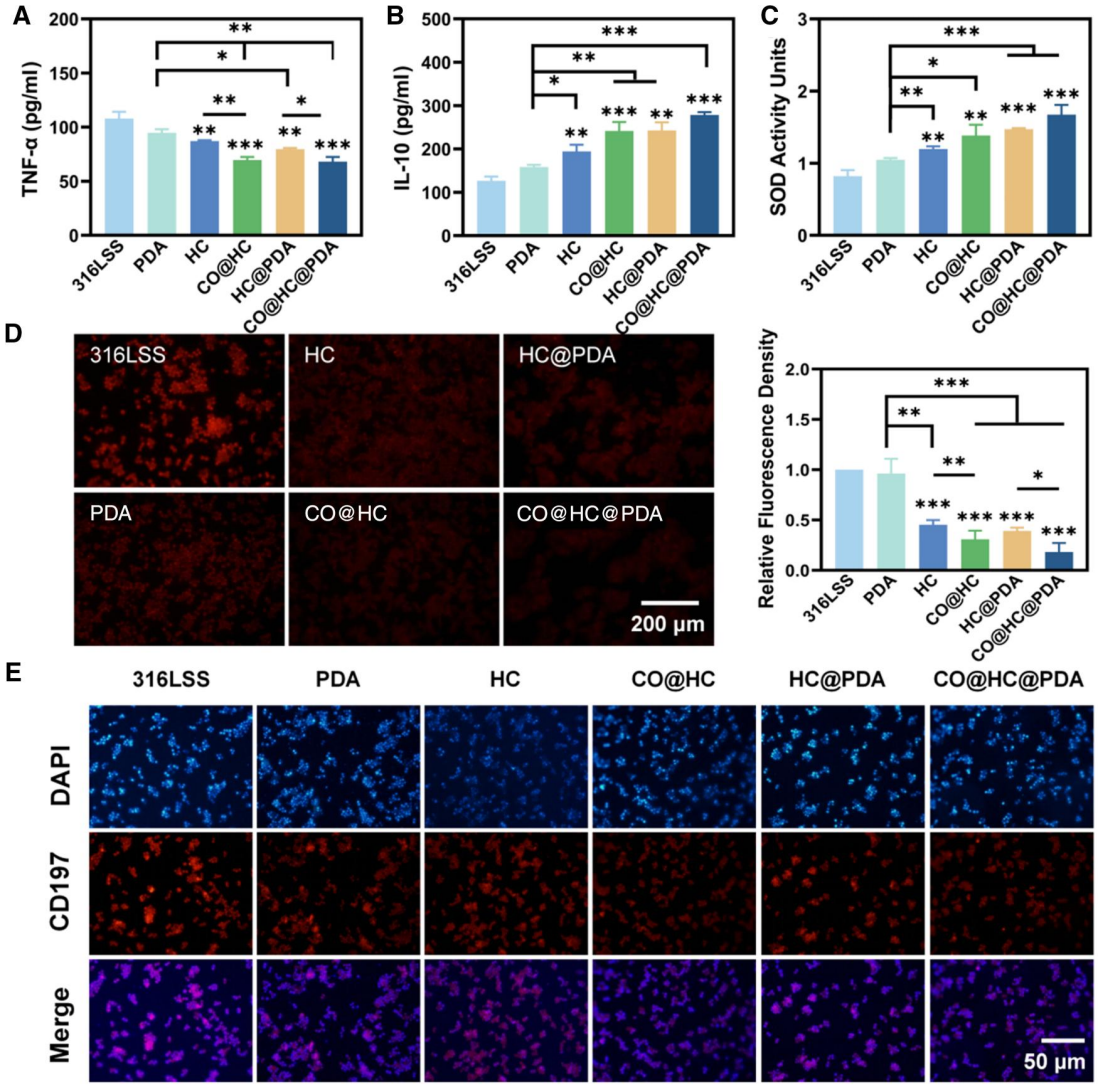
The anti-inflammatory function of CO@HC and CO@HC@PDA *in vitro*. (**A**) TNF-α and (**B**) IL-10 concentration quantification of macrophages co-cultured with samples. (**C**) SOD enzyme activity unit assay. (**D**) Fluorescence images of DHE-stained macrophages and fluorescence intensity quantification results. (**E**) Immunophenotypic analysis of MA. Means ± standard deviation (*n* = 3), *P* < 0.05 (*), *P* < 0.01 (**), and *P* < 0.001 (***).

The LPS-induced macrophage inflammation model was chosen to assess the anti-inflammatory properties of CO@HC and CO@HC@PDA. After a 24-h coculture with LPS-induced macrophages, the supernatants were collected for the analysis of inflammatory factors. Typical proinflammatory factor TNF-α and anti-inflammatory factor IL-10 were specifically examined. The expression of TNF-α in HC, CO@HC, HC@PDA and CO@HC@PDA groups was significantly reduced (Figure 5A). It showed significant decrease of TNF-α expression in HC and HC@PDA compared with 316LSS. It can be attributed to excellent antioxidant capacity of PDA and EGCG which then reduced the inflammatory response [25, 33]. In particular, the TNF-α expression was notably decreased in the CO@HC and CO@HC@PDA groups, suggesting a superior inhibitory effect of CO release on the inflammatory response. Meanwhile, the IL-10 expression was examined (Figure 5B). Significant increases in IL-10 expression were observed in the HC, CO@HC, HC@PDA and CO@HC@PDA groups compared to 316LSS and PDA. Moreover, IL-10 expression was notably higher in the CO@HC and CO@HC@PDA groups than those free of CO loading, suggesting that enhanced CO release contributed to elevated IL-10 levels. In summary, the CO@HC@PDA demonstrated remarkable anti-inflammatory effects by combining MPN@Hb-NPs and CO. Thanks to the integration of MPN@Hb-NPs and CO, the TNF-α expression was notably decreased, while the IL-10 levels were high in both the CO@HC and CO@HC@PDA groups, suggesting remarkable anti-inflammatory efficacy of the both groups.

SOD is an important antioxidant enzyme, which is mainly responsible for scavenging superoxide anion ($O_{2}^{-}$) produced in the cell and acting as an antioxidant. $O_{2}^{-}$ produced during inflammation will trigger oxidative stress, leading to oxidative damage in cells. Results showed the increase of SOD enzyme activity of macrophages under inflammatory conditions co-culture with HC, CO@HC, HC@PDA and CO@HC@PDA compared with 316LSS and PDA (Figure 5C). The SOD activity of CO@HC@PDA groups was the highest, indicating it regulated the behaviors of macrophage and increased its antioxidant ability.

ROS are a class of highly reactive and oxidizing molecules produced in cells, including nitric oxide, $O_{2}^{-}$ and H_2_O_2_. In LPS-stimulated cells, a bright red fluorescence from the ROS indicator (DHE) was evident, indicating a substantial generation of ROS due to LPS stimulation. Conversely, the CO@HC and CO@HC@PDA-treated groups exhibited weaker red fluorescence (Figure 5D), suggesting a reduction in ROS levels.

Macrophage phenotypic markers can more clearly reflect the polarization state of macrophages under inflammatory conditions, and the macrophage M1 phenotype was labeled with CD197, as shown in Figure 5E. It has been documented that PDA has the ability to inhibit the transformation of macrophages into the M1 phenotype [33]. In the present study, it also showed CD 197 expression of macrophages on PDA was lower than that of 316LSS. Meanwhile, CD 197 expression of macrophages on CO@HC and CO@HC@PDA was further attenuated. It indicated CO release inhibited macrophage toward the M1 pro-inflammatory phenotype and thus modulate inflammation.

### *In vitro* evaluation of injured endothelial cells in a simulated ROS-containing microenvironment

**Figure 6.**
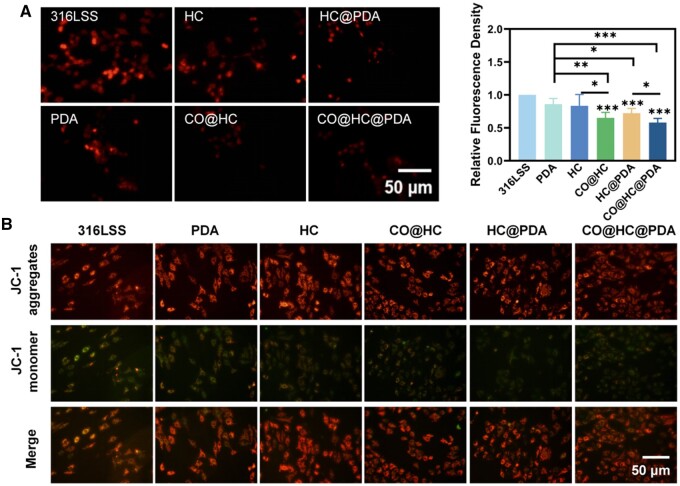
The anti-apoptotic function of CO@HC and CO@HC@PDA *in vitro*. (**A**) Fluorescence images of DHE-stained HUVECs and fluorescence intensity quantification results. (**B**) Fluorescence images of Aggregate JC-1/monomeric JC-1 in HUVECs. Means ± standard deviation (*n* = 3), *P* < 0.05 (*), *P* < 0.01 (**), and *P* < 0.001 (***).

Inflammatory conditions are accompanied by oxidative stress, and an *in vitro* oxidative stress model was established using hydrogen peroxide to stimulate endothelial cells. Under oxidative stress conditions, ROS production increases and exceeds the normal scavenging capacity of cells, leading to oxidative damage. After treatment with H_2_O_2_, the intracellular ROS level was evaluated. Notably, the HC, CO@HC and HC@PDA groups exhibited weak red fluorescence compared to that of SS (Figure 6A). It was the weakest of CO@HC@PDA group indicating that it had the strongest antioxidative ability.

Mitochondrial membrane potential is a crucial marker for detecting early apoptosis. Red fluorescence is produced by JC-1 aggregating within the mitochondrial matrix at high membrane potential. Green fluorescence is produced when JC-1 is unable to assemble and aggregate at low membrane potential staying as a monomer. Higher mitochondrial membrane potential represents more healthy cell. It is obvious that there is a significant increase in the mitochondrial membrane potential level in the HC, CO@HC, HC@PDA and CO@HC@PDA groups when compared to the 316LSS group (Figure 6B), suggesting PDA, MPN and CO all were beneficial to resisting oxidative stress.

### *In vivo* evaluation of the inflammatory modulation function of the CO@HC@PDA

**Figure 7.**
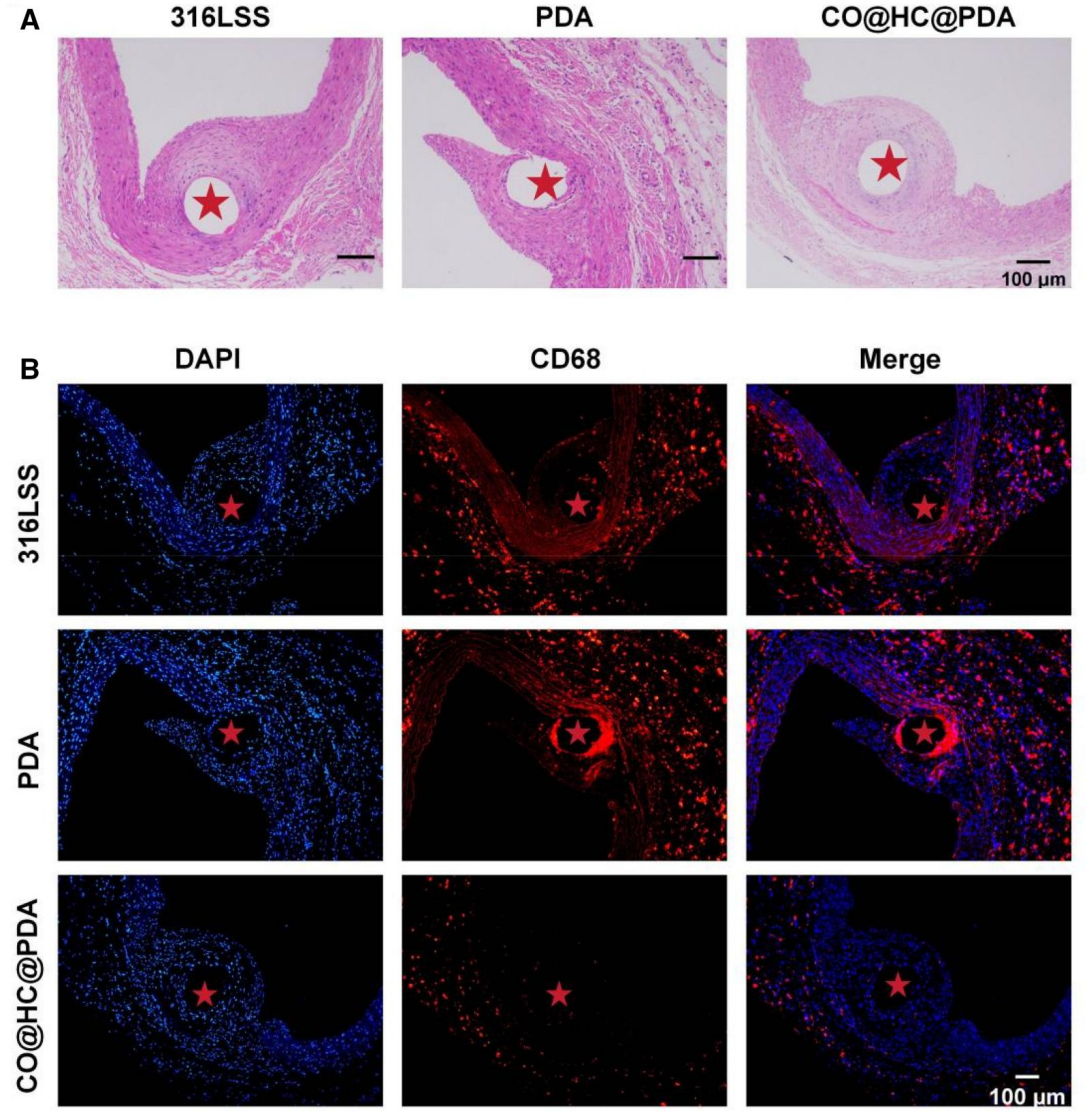
*In vivo* Sprague-Dawley rats implantation results. (**A**) Hematoxylin and eosin-stained after implantation for 30 days. (**B**) Images of CD68 immunofluorescence and nuclei were stained with DAPI.

The efficacy of CO@HC@PDA was evaluated by *in vivo* implanting. Rats were anesthetized and the prepared samples were implanted into the abdominal aorta. The implant process and materials injured vascular wall tissues, and neointimal which encapsulated the implant materials after 30 days was evaluated. HE staining results showed that the 316LSS, PDA and CO@HC@PDA samples were all covered by neointimal tissue after 30 days of implantation (Figure 7A). For the 316LSS and PDA samples, there was a large amount of dark blue tissue with fibrin proximal the samples, which could be mainly an inflammatory response caused by monocytes infiltration and thrombus formation. For the CO@HC@PDA samples, it showed the cell nuclei in the neointimal tissue were evenly distributed and densely organized, and the monocyte infiltration was significantly reduced.

CD68 is the marker of the proinflammatory macrophage. In addition, immunofluorescence staining of CD68 was conducted for exploring inflammatory response of the infiltrated macrophage. Positive and high expression of CD68 was seen on the 316LSS and PDA samples, while that of CO@HC@PDA significantly decreased (Figure 7B). The results confirmed that CO release inhibits macrophage polarization toward the M1 pro-inflammatory phenotype and modulated inflammation.

## Discussion

The gas signaling molecule CO has been shown to modulate inflammation, especially chronic inflammation [34]. However, CO release duration of CORMs is very fast, lasting only a few hours. The iron MOFs MIL-88B and NH_2_-MIL-88B prepared by Ma *et al*. [9] had CO release durations of only 3 and 5 h. Based on improvement of CO duration, our group synthesized MIL-100(Fe), which has a CO release duration of 24 h [10]. However, the Fe content of MIL-100(Fe) is >5%, which may cause the Fenton reaction and produce harmful • OH due to the high Fe content. In this work, the prepared MPN@Hb-NPs have a lower iron content of 0.05% and simultaneously have antioxidant properties with free radicals scavenging (Figure 3C and D and Supplementary Figure S3D). Pan *et al*. [35] designed a CO-releasing coating based on carboxymethyl chitosan-functionalized graphene oxide, with a CO release duration that can reach up to 7 days. In their study, the loading amount of CO was controlled by adjusting the amount of added CO donor CORM401. CORM401 is a manganese carbonyl compound, and the potential risk of manganese ion residue may exist when the donor was added with a large amount.

During the implantation of cardiovascular devices, vascular damage leads to inflammation and oxidative stress. To alleviate inflammation and oxidative stress, a novel biomimetic coating was developed by incorporating green tea polyphenol EGCG and gaseous signaling molecule CO into the surface. EGCG, the predominant and highly effective polyphenol found in green tea, exhibits the capability to stimulate antioxidant enzymes, reduce oxidative stress and inflammation. Moreover, it has the potential to inhibit or scavenge ROS, as well as transfer electrons to free radicals, thus preventing cells from damage [23, 25]. As showed in the results, intracellular ROS decreased (Figures 5D and 6A) and TNF-α expression (Figure 5A) on EGCG-containing coatings, while IL-10 level increased (Figure 5B). It showed CO release had a significant influence on macrophage behavior modulation. It inhibited macrophage polarization toward the pro-inflammatory phenotype M1 (Figure 5E) with decreased TNF-α and increased IL-10 expression (Figure 5A and B).

Normal vascular endothelial cells are crucial for preserving vascular balance and averting atherosclerosis through the management of vascular tone, thrombosis prevention and inflammation regulation. Recent studies highlight endothelial cell apoptosis as the primary trigger for atherosclerosis onset. In addition, the endothelial cells apoptosis prompted by inflammation factors and ROS will reduce the longevity of cardiovascular implants. The decrease in mitochondrial membrane potential is a hallmark event of early apoptosis in cells [36]. It showed the CO@HC@PDA coating is beneficial for keeping mitochondrial membrane potential of endothelial cells and thus it has the ability to prevent endothelial cell from apoptosis under oxidative stress (Figure 6B).

## Conclusion

In this work, a hemoglobin/EGCG-containing functional coating was prepared which had abilities not only CO loading but also antioxidant properties. Under inflammatory microenvironment, the CO@HC@PDA coating modulate macrophage behaviors by decreasing pro-inflammatory factor TNF-α expression, increasing the inflammation-suppressing factor IL-10 expression, inhibiting the M1 polarization and down-regulating the intracellular ROS. Under the oxidative stress microenvironment, the CO@HC@PDA coating reduced endothelial cells apoptosis, and down-regulated the intracellular ROS. The results of abdominal aortic implantation experiments in rats confirmed that the CO@HC@PDA coating modulated inflammation and inhibited macrophages toward the M1 pro-inflammatory phenotype polarization.

## Supplementary Material

rbae148_Supplementary_Data
