# Supplementary material for: CO-loaded hemoglobin/EGCG nanoparticles functional coatings for inflammation modulation of vascular implants
Source: Regen Biomater. 2024 Dec 20;12:rbae148. doi: 10.1093/rb/rbae148 (PMC11781197; doi:10.1093/rb/rbae148)
Supplement: rbae148_Supplementary_Data [file rbae148_supplementary_data.docx]

# Supporting Information

# CO-loaded Hemoglobin/EGCG Nanoparticles Functional Coatings for Inflammation Modulation of Vascular Implants

Sui Wu^b,c^, Ruichen Dong^a,b^, Yinhong Xie^b,c^, Wenhao Chen^a,b^, Wei Liu^a,b^, Yajun Weng^a,b *^

***AUTHOR ADDRESS***

a Institute of Biomedical Engineering, College of Medicine, Southwest Jiaotong University, Chengdu 610031, Sichuan, China

b Key Laboratory of Advanced Technologies of Materials, Ministry of Education, Southwest Jiaotong University, Chengdu 610031, China

c School of Materials Science and Engineering, Southwest Jiaotong University, Chengdu 610031, China

*** Corresponding author**

Email address: wengyj7032@swjtu.edu.cn

## The hydrodynamic diameter, PDI, and ζ-potential results of Hb-NPs and MPN@Hb-NPs


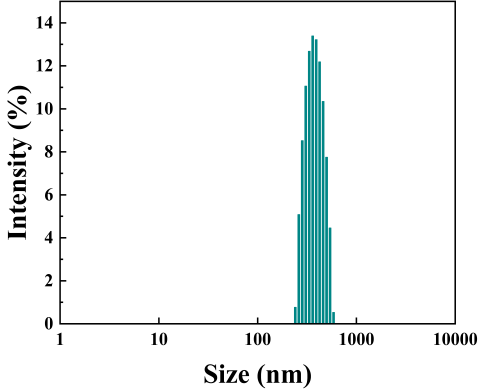


**Figure S1.** Size distribution of MPN@Hb-NPs.

**Table S1.** Hydrodynamic diameter, PDI, and ζ-potential results of Hb-NPs and MPN@Hb-NPs.

|  | Size（nm） | PDI | ζ-potential(mV) |
| --- | --- | --- | --- |
| Hb-NPs | 482 | 0.20 | 4.89 |
| MPN@Hb-NPs | 422 | 0.19 | 19.60 |

## The Fe2p and S2p XPS high resolution spectrum and Elemental analysis of MPN@Hb-NPs


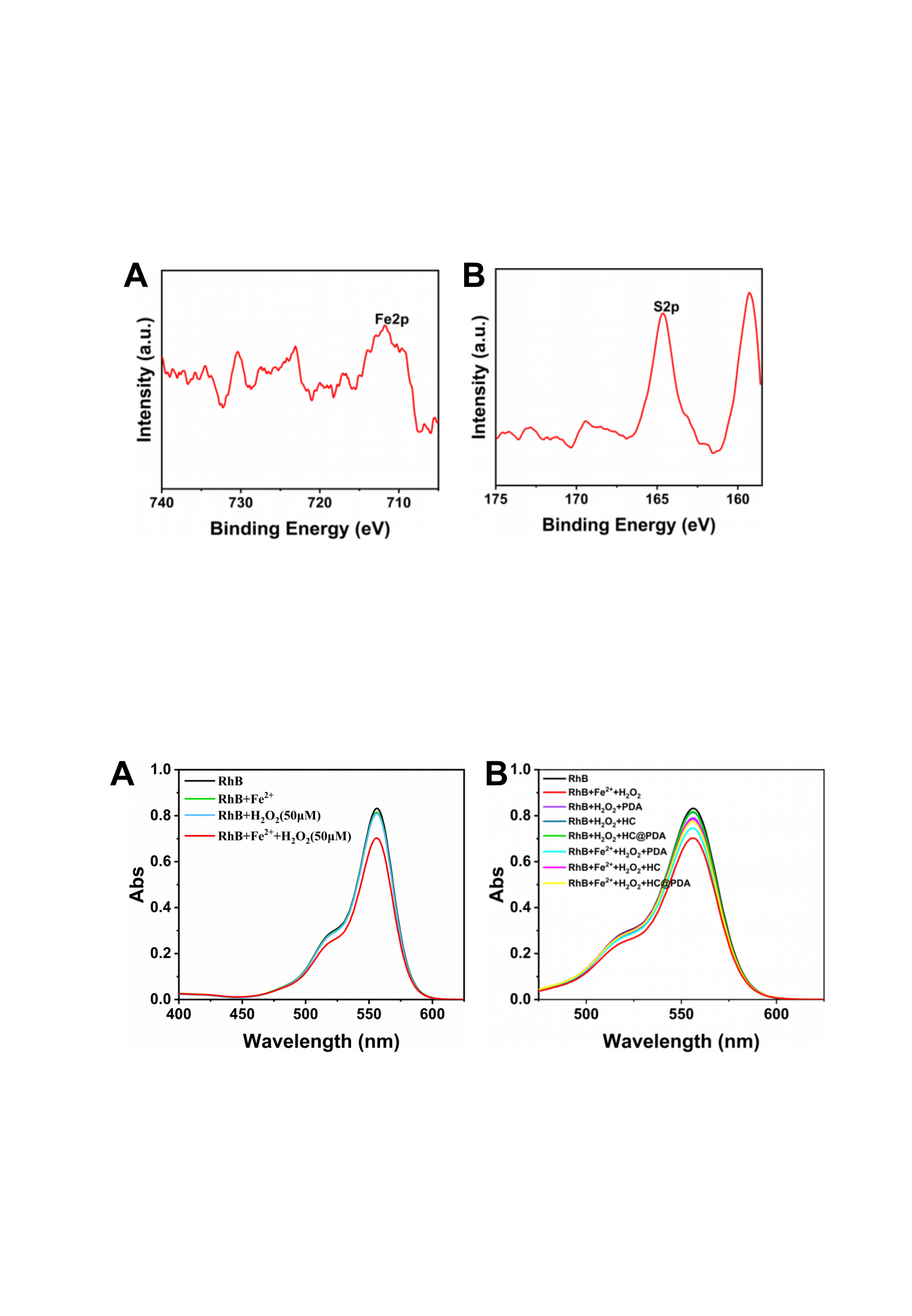


**Figure S2.** The Fe2p and S2p XPS high resolution spectrum of MPN@Hb-NPs.

**Table S2.** Elemental analysis of MPN@Hb-NPs.

| C | Fe | N | O | S |
| --- | --- | --- | --- | --- |
| 72.05 | 0.50 | 8.63 | 18.20 | 0.63 |

## CO Release and DPPH• Scavenging of MPN@Hb-NPs

**Preparation of MPN@BSA-NPs:** 100 mg of BSA was dissolved in 2 mL of ultrapure water. While stirring, 8 mL of ethanol was added gradually. The mixture was stirred for 24 hours. Following the same procedure as for MPN@Hb-NPs, an equal volume and concentration of EGCG and Fe^3+^ solution were added. The mixture was centrifuged at 8000 rpm for 5 minutes and washed three times with deionized water. Finally, it was freeze-dried.

**Preparation of CO@MPN@BSA-NPs:** 1 mg of freeze-dried MPN@BSA-NPs was weighed and added into a 5 mL centrifuge tube. The CO loading method was consistent with that of CO@MPN@Hb-NPs.

**Preparation of MPN (EGCG-Fe^3+^-NPs):** EGCG (10 mg/mL) was dissolved in Tris buffer solution and mixed with Fe^3+^ (10 mg/mL) in a volume ratio of 4:1. The procedure was consistent with that used for MPN encapsulation of hemoglobin. The mixture was centrifuged at 12000 rpm for 5 minutes, washed three times with ultrapure water, and subsequently freeze-dried.

**Preparation of CO@MPN:** 1 mg of freeze-dried MPN was weighed and added into a 5 mL centrifuge tube. The CO loading method was consistent with that of CO@MPN@Hb-NPs.


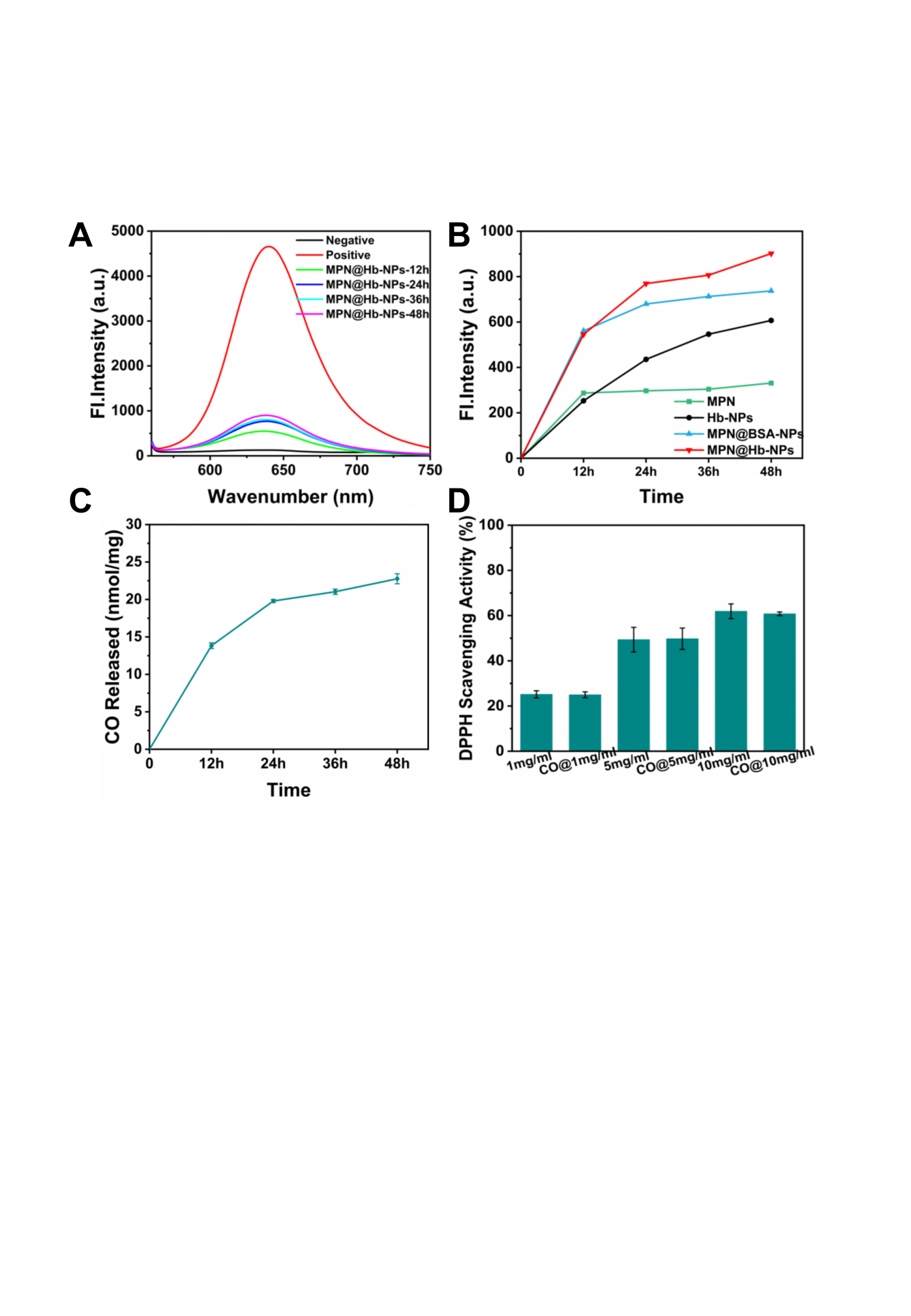


**Figure S3.** CO release from the samples was detected using a 1-Ac fluorescent probe. (A) Fluorescence plots of 1-Ac detection of CO release from negative control, positive control, and MPN@Hb-NPs. (B) Fluorescence intensity of 1-Ac detection of CO release from MPN, Hb-NPs, MPN@BSA-NPs, and MPN@Hb-NPs at 12 h, 24 h, 36 h, and 48 h, respectively. (C) CO release from MPN@Hb- NPs plots of total CO release at different times.(D) DPPH• scavenging rate for MPN@Hb-NPs and CO@MPN@Hb-NPs with different concentrations.

## Fe content of HC and HC@PDA.


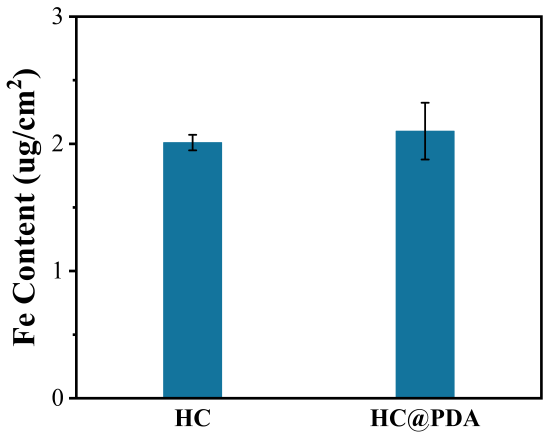


**Figure S4.** Fe content of HC and HC@PDA

## The XPS spectra of HC and HC@PDA


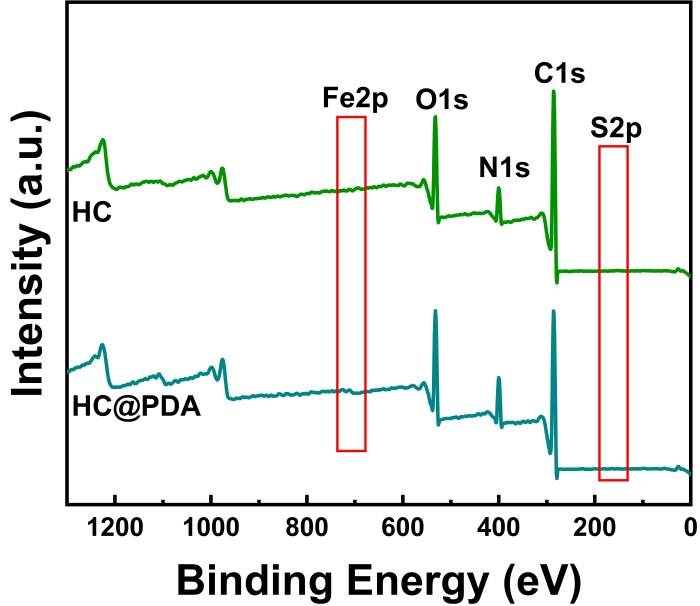


**Figure S5.** The XPS spectra of HC and HC@PDA.

**Table S3.** Elemental analysis of HC and HC@PDA

|  | C | N | O | Fe | S |
| --- | --- | --- | --- | --- | --- |
| HC | 69.12 | 12.31 | 18.08 | 0.33 | 0.16 |
| HC@PDA | 74.45 | 9.07 | 16.20 | 0.17 | 0.11 |
